# Seasonal Changes in Psychomotor Abilities of Male Handball Players

**DOI:** 10.3390/brainsci16030338

**Published:** 2026-03-21

**Authors:** Maciej Śliż, Wojciech Paśko, Francisco Martins, Rafał Krupa, Élvio Rubio Gouveia, Hugo Sarmento, Krzysztof Przednowek

**Affiliations:** 1 Faculty of Physical Culture Sciences, Collegium Medicum, University of Rzeszow, 35-959 Rzeszow, Poland; wopasko@ur.edu.pl (W.P.); krprzednowek@ur.edu.pl (K.P.); 2 Universidade de Coimbra, CIPER, Faculdade de Ciências do Desporto e Educação Física, 3004-504 Coimbra, Portugal; joao.martins@staff.uma.pt (F.M.); hg.sarmento@gmail.com (H.S.); 3 Department of Physical Education and Sport, University of Madeira, 9020-105 Funchal, Portugal; erubiog@staff.uma.pt; 4 LARSYS, Interactive Technologies Institute, 9020-105 Funchal, Portugal; 5 Zespół Szkolno-Przedszkolny nr 20, ul. Karpnicka 2, 54-061 Wrocław, Poland; rkrupa1994@gmail.com; 6 CIPER, Faculdade de Motricidade Humana, Universidade de Lisboa, Cruz-Quebrada-Dafundo, 1495-751 Lisbon, Portugal

**Keywords:** psychomotor abilities, handball, reaction time, competition season, position on the court

## Abstract

**Highlights:**

**What are the main findings?**

- Full-season competition leads to a significant reduction in reaction times for choice reaction, hand–eye coordination, and spatial orientation tests among professional handball players.
- While reaction speed improves, motor abilities parameters tend to deteriorate after the season, evidenced by increased movement time in simple and choice reaction tasks.

**What are the implications of the main findings?**

- The findings suggest that seasonal fatigue and training adaptations create a unique psychomotor profile that requires monitoring to balance improved cognitive processing with declining motor execution.
- Position-specific differences in psychomotor evolution throughout the season indicate a need for individualized training and recovery strategies to maintain peak performance across all court roles.

**Abstract:**

**Background/Objectives:** Reaction time, hand–eye coordination, spatial orientation, and attention play a key role in handball, which is characterized by high intensity as well as high cognitive and motor demands. The level of these abilities may change during the season, potentially reflecting training adaptations and increasing physical fatigue. The aim of the study was to compare the level of psychomotor abilities in professional handball players before the start of the competition round and after the end of the league season. The study included 77 handball players playing in the Polish Handball Super League (average age: 25.6 ± 5.2 years). The players were divided according to position: pivot, center, and wing. **Methods:** Psychomotor abilities were assessed using the Test2Drive computer system, employing tests of simple and choice reaction time, eye–hand coordination, spatial orientation, perception and attention, and movement anticipation. **Results:** At the end of the season, a statistically significant reduction in reaction time was observed in the choice reaction (*p* = 0.001), eye–hand coordination (*p* = 0.002), and spatial orientation tests (*p* = 0.003). In terms of motor skills, an increase in time was observed in the SIRT test (*p* = 0.003), CHORT (*p* = 0.005) and HECOR (*p* = 0.011) tests, while the time in the PUT test was shortened for both neutral (*p* = 0.002) and critical (*p* = 0.025) stimuli. Positional analysis showed that after the season, the pivot player achieved higher effectiveness in the CHORT test than the wing player (*p* = 0.020). Additionally, statistically significant differences were observed for correct responses in the SPANT test (*p* = 0.032). In terms of correct answers in the PAMT test, the pivot player had the lowest effectiveness. **Conclusions:** Participation in the full season of competition coincided with significant changes in the psychomotor profile of handball players, with a simultaneous improvement in reaction speed and deterioration in movement time parameters.

## 1. Introduction

The significance of psychomotor abilities in male handball players extends across the entire competitive season, influencing both performance and the developmental trajectory of athletes in this high-intensity sport [1,2]. Psychomotor abilities, including coordination, reaction time, and agility, are essential for effective gameplay in handball, demanding that athletes exhibit high levels of skill both physically and mentally [3,4,5]. These abilities can be fundamental to success in handball [6,7]. Studies illustrate that well-developed psychomotor abilities enhance individual performance and contribute to team dynamics and efficiency on the court [8,9].

Research by Massuça et al. [3] reveals that elite handball players possess a distinct advantage in terms of task orientation, motivation, and skill mastery, which are critical components linked to psychomotor performance. These attributes enhance players’ intrinsic motivation and their ability to persist through the challenges faced during a season. Given handball’s demanding physical and tactical context, the ability to execute complex movements and strategies under pressure is vital. Psychomotor abilities are thus a foundational element of competitive resilience, allowing players to adapt to various game scenarios effectively [10,11]. Research by Przednowek et al. [10] identifies key psychomotor abilities in professional handball players, emphasizing how these skills can vary significantly depending on factors such as league classification, position played, and training experience. Their findings suggest that elite athletes demonstrate superior psychomotor capabilities compared to non-players, highlighting inherent differences fostered through specialized training and competition participation [10]. This supports the notion that the competitive environment enhances these abilities, which can be reassessed after the season ends.

Furthermore, psychomotor performance can be significantly influenced by targeted training programs. For instance, Hermassi et al. [4] demonstrate the effectiveness of plyometric training in enhancing both repeated sprint ability and overall leg power in handball players, suggesting that systematic training positively contributes to psychomotor development. In addition, Krawczyk et al. [12,13] reported a significant correlation between higher levels of psychomotor abilities and the effectiveness of goalkeepers in high-stakes game scenarios, indicating that consistent practice and competition refine these skills over time. In terms of psychological factors, Muntianu et al. [14] investigate the correlation between various psychological traits and psychomotor abilities in junior handball players, elucidating how mental skills such as concentration, emotional control, and coping strategies inform an athlete’s physical performance. Training methods that include specific assessments of psychomotor abilities, such as reaction times and agility, should therefore be routinely employed not only before the onset of the competitive season but also at its conclusion. This will help in understanding the degree of skill enhancement and potential deficits that may arise from competitive play, ultimately informing future training strategies tailored to individual athlete needs [12,14].

Moreover, Bresciani et al. [15] conducted an extensive longitudinal study surveying biological and psychological measures throughout a competitive season, noting the influence of mood and stress on performance. Their findings indicated that fluctuations in biological markers, such as inflammation and oxidative stress, were observable in players during intensive training and could impact psychomotor capabilities. This emphasizes the need for coaches and trainers to consider psychological well-being as a vital aspect of player development during the season. As the season progresses, maintaining a strong foundation in psychomotor abilities becomes increasingly important. Research findings support the integration of diverse training approaches, fostering psychological resilience among players to optimize performance levels [16,17]. Overall, the effectiveness of male handball players is invariably linked to their psychomotor capabilities, emphasizing the need for sustained attention to these skills throughout the competitive season [14,18].

In summary, most of the scientific papers to date on the assessment of psychomotor abilities has focused mainly on comparing athletes of different skill levels or with a control group of untrained individuals [1,10]. Therefore, monitoring psychomotor parameters throughout the league season, where the level of these skills may change due to training adaptation, cumulative competition stress, and increasing physical fatigue, is very necessary. In addition, handball is a highly specialized team sport that places different psychomotor demands on pivots, center players, and wingers. Previous reports suggested that players in different positions differ in terms of reaction time, motor skills, and decision-making, but it was not explained how these profiles evolve during the league season. Therefore, the aim of the study was to (i) compare the levels of psychomotor abilities in professional male handball players before and after a competitive season; (ii) examine the differences in these abilities according to specific playing positions; and (iii) evaluate the relationship between psychomotor abilities, training seniority, and Super League experience.

## 2. Materials and Methods

This investigation involved 77 professional male handball players (25.6 ± 5.2 years old; 10.0 ± 5.9 years of seniority training) currently active in the Polish Handball Super League or Central Men’s League (Table 1). They were divided into three specific playing positions: pivot players (*n* = 15), center players (*n* = 38), and wing players (*n* = 24). The inclusion criteria were as follows: (a) each player was of legal age; (b) the study only included male players; (c) a minimum of 2 years of experience playing at the senior level; (d) players had to be free of any injuries; (e) players had to play in one of three positions (pivot, center, or wing player). The study had very high statistical power (1 − β = 0.99), calculated post hoc, which means that the sample size was fully sufficient to detect the analyzed effect. All procedures implemented in this study were approved by the Ethics Committee of the University of Madeira (151/CEUMA/2024, 21 November 2024), and written informed consent was obtained from all participants.

### 2.1. Body Composition

Height was measured to the nearest 0.1 cm using a stadiometer (SECA 213, Microgate, Hamburg, Germany). Body composition was assessed using hand-to-foot bioelectrical impedance analysis (InBody 770, InBody, Cerritos, CA, USA). On the platform, participants stood barefoot with their arms positioned at a 45-degree angle from their trunk and their feet on the designated spots. During the measurement, the participants were fasting and using only their underwear. The assessment was conducted in the early morning in laboratory settings. Body mass (kg), body fat (kg), body fat percentage (%), fat-free mass (kg), and body mass index (kg/m^2^) were retained for analysis.

### 2.2. Psychomotor Abilities Assessment

**Figure 1:**
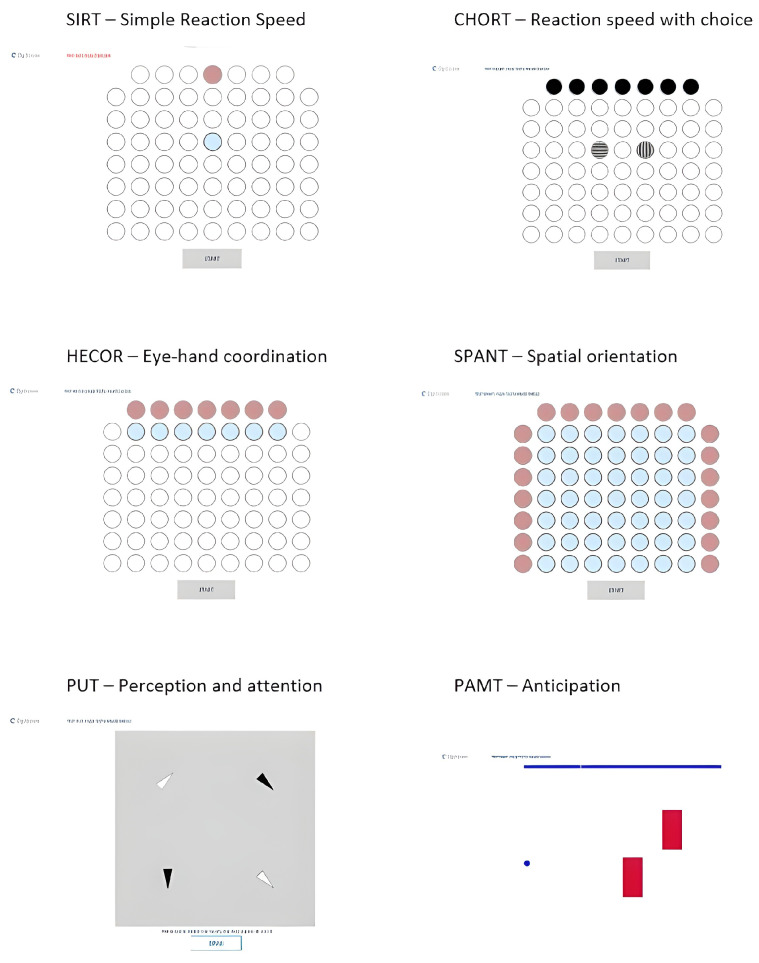
Test2Drive tests: SIRT; CHORT; HECOR; SPANT; PUT; PAMT.

The Test2Drive computer system was used to assess psychomotor abilities [19]. The study employed six tests presented in Figure 1 to assess the following:

(a) Simple reaction speed (SIRT)—The test assessed the speed of response to a visual stimulus. The task was to move the index finger from the “START” field to the response field (blue dot) as quickly as possible after the appearance of the visual stimulus (red dot). The reaction field and the visual stimulus were in the same location throughout the test. After reacting as quickly as possible to the visual stimulus, the finger returned to the “START” field and waited for the next stimulus. The reaction time and movement time were analyzed. The reaction time (RT) was the time from the appearance of the visual stimulus until the “START” field was released. The movement time (MT) indicates the time from releasing the “START” field to clicking the reaction field.
(b) Reaction speed with choice (CHORT)—The test assessed the speed and adequacy of responses to visual stimuli. The test consisted of responding appropriately to a given stimulus. Vertical, horizontal, or diagonal lines appeared in the black fields at the top of the screen. In the middle of the screen were response fields (vertical and horizontal lines). When a visual stimulus (vertical or horizontal lines) appeared, the subject moved their finger from the “START” field to the appropriate response field as quickly as possible. When diagonal lines appeared, the subject had to remain unresponsive. The number of correct answers, reaction time, and movement time were analyzed.
(c) Eye–hand coordination (HECOR)—The test assessed eye–hand coordination by analyzing reaction speed to stimuli. At the top of the screen were bright red fields in which a visual stimulus (dark red dot) appeared at random, and the subject had to react as quickly as possible by moving their finger from the “START” field to the appropriate reaction field located below the visual stimulus. The reaction time and movement time were analyzed.
(d) Spatial orientation (SPANT)—The test assessed eye–hand coordination using spatial information. On the sides of the screen, there were bright red fields on which two visual stimuli appeared simultaneously. One visual stimulus appeared at the top of the screen, while the other appeared on the left side of the screen. The task of the test subject was to move their finger from the “START” field to the reaction field, which is the intersection of the two visual stimuli. The number of correct answers, reaction time, and movement time were analyzed.
(e) Perception and attention (PUT)—The test assessed visual attention. White and black triangles appeared on the screen, which could be arranged vertically, horizontally, or diagonally. The task of the test subject was to identify and select the vertical black triangle as quickly as possible. If there was no such triangle on the board, the participant selected the “NONE” field. Each response should be made as quickly as possible. The test took into account reaction speed (PUT n and PUT k). PUT n referred to the reaction speed when a vertical black triangle was on the board, while PUT k referred to the reaction speed when the indicated figure was not present and the “NONE” button was selected. In addition, the number of correct answers (cr) was analyzed.
(f) Anticipation (PAMT)—The test assessed movement prediction in a complex and dynamic situation. The task was to move the ball to the other side of the monitor to avoid colliding with two red rectangles that were moving up and down. The ball moved in a straight line horizontally, and its speed was visible on the upper blue bar. When the participant decided that there would be no collision at a given moment, they clicked on the ball, which then began to move to the other side of the screen. The number of correct answers (cr) was taken into account for the analysis.

The tests were conducted in a closed room without the presence of anyone else. The temperature during the tests was 19 °C. The measurement was performed in a standing position, with the monitor positioned at the height of the participant’s hips. Each test was performed by the participant using the index finger of their dominant hand. Each test was preceded by a trial run to allow the participants to familiarize themselves with the test procedure. All tests were performed in the same order.

### 2.3. Statistical Analysis

In order to characterize the group and evaluate selected psychomotor abilities for the analysis of results, basic statistical measures such as the mean, standard deviation, first quartile, median, third quartile, difference, and interquartile range were used. In addition, the normal distribution of the studied variables was verified using the Shapiro–Wilk test. The differences between the levels of psychomotor abilities before and after the competition round were assessed using the Wilcoxon test, as the variables deviated from the normal distribution. The effect size for the Wilcoxon test was calculated using the rank-sum correlation coefficient. Additionally, the level of psychomotor abilities was compared according to position using the Kruskal–Wallis test, while the effect size was calculated using $\eta^{2}$. Moreover, a post hoc analysis was conducted using pairwise comparisons with the Dwass–Steel–Critchlow–Fligner (DSCF) method. Correlation analysis was performed using Spearman’s rank correlation. All analyses were conducted using Jamovi version 2.6.44 (The Jamovi Project, Sydney, Australia). The test power analysis was performed using G*Power 3.1.9.7. The significance level was set at p≤0.05.

## 3. Results

The assessment of psychomotor abilities before and after the competition round is presented in Table 2. Statistically significant differences were observed for reaction time in the CHORT, HECOR, and SPANT tests. It was shown that after the competition round, athletes exhibited shorter reaction times in each of these tests. In the CHORT test, reaction time was 45 ms shorter, with an effect size of r = 0.44. In the HECOR test, reaction time decreased by 15 ms after the round, with an effect size of r = 0.42. In the SPANT test, reaction time was 16 ms shorter after the competition round, with an effect size of r = 0.39.

The analysis of movement time before and after the competition round revealed statistically significant differences in the SIRT, CHORT, HECOR, PUT n, and PUT k tests. For the SIRT, CHORT, and HECOR tests, longer movement times were observed after the competition round. In the SIRT test, movement time increased by 15 ms after the round, with an effect size of r = −0.40. In the CHORT test, movement time was 19 ms longer after the round, with an effect size of r = −0.37. A similar increase in movement time was observed in the HECOR test, with an effect size of r = −0.33. After the competition round, shorter movement times were observed in the PUT test for both neutral and critical stimuli. For neutral stimuli, movement time decreased by 185 ms, with an effect size of r = 0.42. For critical stimuli, movement time was 42 ms shorter after the round, with an effect size of r = 0.29.

The analysis of correct responses revealed statistically significant differences only in the SPANT test (*p* = 0.032). Both before and after the competition round, the first quartile, median, and third quartile did not change their values. The effect size for correct responses in the SPANT test was r = −0.34.

In Table 3, an analysis of the level of psychomotor abilities by playing position was conducted. For the Pivot player, statistically significant differences were observed for reaction time in the CHORT and SPANT tests. In both cases, shorter reaction times were recorded after the competition round. Reaction time in the CHORT test was 689 ms before the round and 644 ms after the round. The effect size for CHORT RT was r = 0.70. In the SPANT test, it was observed that reaction time was 641 ms before the round and 597 ms after the round. The effect size for SPANT RT was r = 0.77.

The assessment of psychomotor abilities for the center player showed statistically significant differences in movement time in the SIRT, CHORT, and HECOR tests. It was observed that after the competition round, center players demonstrated longer movement times in each of these tests. In the SIRT test, movement time was 178 ms before the round and 190 ms after the round. The effect size for SIRT MT was r = −0.42. In the CHORT test, movement time was 195 ms before the round and increased to 212 ms after the round, with an effect size of r = −0.55. The evaluation of movement time in the HECOR test showed that it was 220 ms before the round and increased to 242 ms after the round. The effect size for HECOR MT was r = −0.49.

The analysis of the wing player revealed statistically significant differences in reaction time in the SIRT, CHORT, and HECOR tests. For SIRT RT, a shorter reaction time was observed after the competition round (327 ms), with an effect size of r = 0.57. In the CHORT test, a shorter reaction time was also recorded after the round (625 ms), with an effect size of r = 0.70. Similarly, in the HECOR test, reaction time was shorter after the competition round (383 ms), with an effect size of r = 0.74. Moreover, statistically significant differences were observed for CHORT cr and PUT n in the wing player. For CHORT cr, it was found that after the competition round, wing players achieved 94% correct responses, whereas before the round, the percentage of correct responses was 98%. The effect size for CHORT cr was r = 0.66. For PUT n, it was observed that after the competition round, wing players achieved shorter movement time (1733 ms), with an effect size of r = 0.59.

The analysis of differences between playing positions revealed statistically significant differences for CHORT cr after the competition round and for PAMT cr before the competition round. For CHORT cr, it was observed that after the competition round, pivot players demonstrated the highest accuracy (100%). Additionally, the post hoc analysis showed statistically significant differences between pivot players and wing players, who had the lowest accuracy (94%). For PAMT cr, it was observed that both center players and wing players achieved 89% correct responses, while pivot players reached 78%. Furthermore, the post hoc analysis also revealed statistically significant differences between wing players and pivot players.

The analysis of the relationship between psychomotor abilities, training experience, and Super League experience was conducted in Table 4. Before the competition round, it was observed that Super League experience was significantly correlated with movement time in the SIRT, CHORT, and HECOR tests. In each case, a positive correlation was found, indicating that the longer the Super League experience, the longer the movement time. The assessment of psychomotor abilities after the competition round showed that training experience was significantly correlated with movement time in the SIRT and HECOR tests. Both correlation coefficients were positive, meaning that the longer the training experience, the greater the movement time in the SIRT and HECOR tests.

## 4. Discussion

The main challenge in modern handball is maintaining optimal psychomotor performance throughout the league season, which can be characterized by high intensity and variable cognitive and physical demands [20]. Psychomotor abilities, defined as the precision and coordination of movements in response to external stimuli, involve the process of selecting and processing information, which allows for the adequate performance of motor tasks during a handball match [10,16]. Therefore, the literature often emphasizes that these functions can be critical to success because they determine the speed of reaction to situations on the court, the accuracy of decisions under time pressure, and the ability to anticipate and predict the opponent’s movements [21,22]. After conducting research and analyzing the data, a difference was observed between reaction time (RT) and movement time (MT). The results indicate that handball players, after a full competition cycle, show significantly shorter reaction times in tests requiring choice (CHORT, *p* = 0.001), eye–hand coordination (HECOR, *p* = 0.002), and spatial orientation (SPANT, *p* = 0.003). At the same time, a prolongation of movement time parameters was noted in the SIRT (*p* = 0.003), CHORT (*p* = 0.005), and HECOR (*p* = 0.011) tests. The improvement in reaction time may suggest that the systematic mental and physical demands of playing at the highest level promote more efficient processing of visual stimuli and faster decision-making. In the literature, this phenomenon is associated with the development of perceptual and cognitive skills that allow elite athletes to “read” the game faster [23]. Therefore, psychomotor abilities can play an essential role in different sports, as various discipline require specific developments in reaction time, suggesting that the context of the competition influences reaction time outcomes [24]. Research has highlighted the nuanced relationship between training, competition, and psychomotor efficiency, showcasing the cognitive mechanisms that athletes leverage in high-pressure environments [25,26]. That is why our investigation aimed to compare the levels of psychomotor abilities, examine the differences in these abilities according to specific playing positions, and evaluate possible relationships between them and training seniority and experience in the Polish Super League. The main results of this study indicate that handball players became faster at reacting to stimuli after completing a full sporting season. Interestingly, their motor execution time (represented in this study as movement time) increased after the season, compared to players’ pre-season performances, particularly in SIRT, CHORT, and HECOR tests. After an entire sports season, the handball players who participated in this study demonstrated significant improvements in their performance, as measured by reaction time (CHORT, HECOR, and SPANT tests). More specifically, when comparing pre-season results with post-season results, a decrease was observed in reaction time in assessments of choice, eye–hand coordination, and spatial orientation. This positive differentiation from the beginning to the end of a sports season, in terms of reaction time, is supported by scientific literature, which has observed significant time reductions in players who underwent intense training/competitions over a prolonged period [27,28,29,30]. These improvements have been associated with fluctuations in competitive stress levels and the development of perceptual and cognitive skills throughout a sports season, which has been linked to faster reading and processing of a set of stimuli, thereby reducing reaction time [31,32,33]. Indeed, research in this area has often highlighted possible relationships between training, competition, and psychomotor efficiency indices, demonstrating the importance of developing cognitive mechanisms throughout the season, particularly in high-pressure situations that require the fastest possible reactions [34]. Although the previous result is somewhat expected, the same positive evolution does not seem to occur in movement time throughout a season at the highest level. Specifically in this study, the movement time at the end of the competitive season, compared to the results achieved in the pre-season, was worse, i.e., higher (CHORT and HECOR tests). The combination of results achieved shows that although reaction time decreased from the beginning to the end of the season, movement time did the opposite, increasing movement time in choice and eye–hand coordination assessments. One of the main hypotheses that may explain these results is that players are subject to high-intensity demands throughout a professional handball season, which can typically increase their neuromuscular fatigue levels and consequently decrease their motor execution speed [35,36].

In addition, these data are strongly corroborated by the correlation analysis we performed later. This showed us that the players in this sample with more experience, training, and competition time in the Polish Super League were those who had slower and, consequently, longer movement times. Following the same line of reasoning as before, more experienced players tend to accumulate greater physical wear and tear because they have already participated in more high-performance sports seasons. One of the hypotheses most supported by the literature is that this could reflect a strategic adaptation, where more experienced handball players prioritize maximum accuracy over movement time, or simply the long-term physical fatigue associated with elite-level competition [37,38,39].

While the learning effect typically improves performance through repeated use of a tool, this study showed inconsistent results. Specifically, reaction time (RT) significantly improved, but movement time (MT) increased during the CHORT and HECOR tests. If these changes were caused only by the learning effect, both parameters should have improved together. The decline in MT suggests that the results reflect actual physiological changes, such as neuromuscular fatigue, rather than just practice.

Interestingly, the results of our study showed different trends for the positions on the field analyzed compared to the psychomotor results achieved at the beginning and end of the studied sports season. For instance, players in the pivot position demonstrated significant improvements in reaction time and reaction speed with the choice (CHORT) and spatial orientation assessments (SPANT), while maintaining 100% accuracy. This superior performance in complex psychomotor tasks may be justified by the specific tactical demands of the pivot position, such as playing in the most congested area of the court, frequently with their back to the goal and surrounded by defenders [10,40]. In contrast, wing players were the only ones to show a significant decrease in simple reaction time (SIRT) assessments. This result may reflect the importance of this skill for this specific position in the field, such as exploiting direct and fast counterattack situations [1,10,40]. Regarding players who operate in the central area of the field and along the first attacking line, we observe a more balanced profile across their psychomotor abilities. However, they have significantly prolonged their movement time from the beginning to the end of the studied season. In this specific case, the high volume of actions and decision-making required to play in the front line of a handball team may be associated with greater physical exhaustion and, consequently, impair the neuromuscular efficiency of players in this area of the field, despite maintaining their reaction speed and game reading [10,41,42]. Overall, the differences inherent to each specific position on the field underscore the need to employ distinct training strategies, recovery methods, physical loads, and mental exercises tailored to each position, based on their tactical function and psychomotor demands during play [20,43,44,45].

Despite the results obtained, this study has also several significant limitations that should be taken into account when analyzing and interpreting the results. Although the number of participants is robust when considering a professional competitive level, the positional distribution results in a small sample for each specific position on the field.

The sample size of n = 15 for the pivot subgroup, although seemingly small in the context of general statistics, is often the limit of recruitment possibilities in competitive sports. In elite handball teams, the number of pivots is naturally smaller than that of playmakers or wingers. In addition, at the highest level of competition, there are an average of two pivot players per team, and there are 13 teams (Orlen Superliga Men’s Handball). Therefore, this is a small group of players who constitute an elite group among handball players.

In addition, several limitations of the present study should be acknowledged. First, the lack of a control group makes it impossible to definitively attribute the observed changes throughout the season to the training process. Second, the absence of objective measures of exertion (e.g., sRPE or playing time) represents a significant limitation of the study. Future research should consider monitoring handball players using a GPS system or applying the session Rating of Perceived Exertion (sRPE) to better assess training load. Finally, future analyses should also take calendar age into account as a potential confounding factor in order to more precisely distinguish the effects of long-term training from those related to the aging process.

Furthermore, psychological variables and objective indicators for measuring stress and fatigue were not considered during either of the two assessment moments or throughout the entire sports season. The implementation of such assessments could provide more profound and more specific insights into the variation in performance in the psychomotor parameters studied.

Finally, the use of a computerized system with Test2Drive, although a highly valid instrument corroborated by the scientific literature for measuring the psychomotor abilities of handball players, does not allow for the global reproduction of the complete and multifactorial environment required for participation in a high-performance handball game.

## 5. Conclusions

In conclusion, significant shifts in psychomotor parameters were observed during the league season in a handball season. On the one hand, although reaction time improves significantly, movement time tends to worsen from the beginning to the end of the season. These changes may be associated with the presence of accumulated physical fatigue. Differentiating between positions on the field, pivots demonstrate a greater ability to react to complex stimuli, while wingers improve their simple reaction speed. Players lining up in the central area of the field were most affected by fatigue related to increased motor execution time. In practical terms, technical teams should consider these aspects when implementing training and recovery protocols, ensuring that the specific physical demands of each position on the field are respected and taken into account. It was noted that monitoring cognitive abilities and reaction time can be an essential index to help optimize training and competition processes throughout a sports season. Future research will benefit from considering the integration of functional field tests and combining them with psychomotor laboratory assessments.

## Figures and Tables

**Table 1:** Characteristics of the study group.

	Pivot Player	Center Player	Wing Player	All
*n*	15	38	24	77
Training seniority	11.0 ± 6.7	9.7 ± 5.7	9.8 ± 5.9	10.0 ± 5.9
Age	27.2 ± 5.9	25.3 ± 4.8	25.1 ± 5.5	25.6 ± 5.2
Body height (cm)	192.5 ± 8.5	191.5 ± 5.8	182.8 ± 4.1	189.0 ± 7.2
Body mass (kg)	108.0 ± 10.6	93.0 ± 10.6	83.9 ± 5.2	93.1 ± 12.4
BMI (kg/m^2^)	29.2 ± 2.4	25.4 ± 2.2	25.2 ± 1.5	26.1 ± 2.6

**Table 2:** Numerical characteristics of psychomotor abilities before and after the round.

Variable	B			A			D	*p*	r
	Q1	Me	Q3	Q1	Me	Q3	(B-A)		
SIRT RT (ms)	325	343	364	318	335	355	8	0.063	0.24
SIRT MT (ms)	161	180	201	167	195	227	−15	0.003 *	−0.40
CHORT RT (ms)	639	682	721	603	637	691	45	0.001 *	0.44
CHORT MT (ms)	173	200	227	190	219	250	−19	0.005 *	−0.37
CHORT cr (%)	92	96	100	92	96	100	0	0.989	0.00
HECOR RT (ms)	386	410	438	377	395	416	15	0.002 *	0.42
HECOR MT (ms)	204	228	257	224	247	273	−19	0.011 *	−0.33
SPANT RT (ms)	557	585	674	507	569	641	16	0.003 *	0.39
SPANT MT (ms)	205	234	256	207	240	267	−6	0.380	−0.12
SPANT cr (%)	90	95	100	90	95	100	0	0.032 *	−0.34
PUT n (ms)	1679	1926	2144	1559	1741	2028	185	0.002 *	0.42
PUT k (ms)	1189	1246	1347	1113	1204	1323	42	0.025 *	0.29
PUT cr (%)	94	96	98	94	96	98	0	0.593	−0.08
PAMT cr (%)	78	83	89	78	83	89	0	0.583	−0.08

B—Before; A—After; SIRT—Simple Reaction Time; CHORT—Choice Reaction Time; HECOR—Hand—Eye Coordination Test; SPANT—Spatial Anticipation Test, PUT—Perception and Attention Test; PAMT—Anticipation Test; RT—Reaction Time; MT—Movement Time; cr—correct responses; n—neutral visual stimuli; k—critical visual stimuli; Q1—first quartile; Me—median; Q3—quartile three; D(B-A)—difference between before and after; *p*—probability of testing (Wilcoxon test); r—rank-sum correlation coefficient; *—statistical significance.

**Table 3:** Numerical description of psychomotor abilities by position.

Variable		Pivot Player			Center Player			Wing Player			$p_{\mathrm{kw}}$	$\eta^{2}$	p-h
		Me ± IQR	$p_{w}$	r	Me ± IQR	$p_{w}$	r	Me ± IQR	$p_{w}$	r			
SIRT RT (ms)	B	346 ± 43	0.426	0.24	332 ± 44	0.489	0.15	356 ± 35	0.013 *	0.57	0.087	0.06	
	A	347 ± 50			334 ± 23			327 ± 51			0.158	0.05	
SIRT MT (ms)	B	190 ± 57	0.107	−0.48	178 ± 48	0.049 *	−0.42	179 ± 38	0.103	−0.38	0.436	0.02	
	A	221 ± 46			190 ± 61			191 ± 53			0.206	0.04	
CHORT RT (ms)	B	689 ± 66	0.018 *	0.70	657 ± 99	0.517	0.14	693 ± 68	0.003 *	0.70	0.069	0.07	
	A	644 ± 46			638 ± 128			625 ± 72			0.467	0.02	
CHORT MT (ms)	B	221 ± 61	0.379	−0.27	195 ± 37	0.010 *	−0.55	195 ± 56	0.153	−0.34	0.153	0.05	
	A	220 ± 58			212 ± 70			220 ± 46			0.545	0.02	
CHORT cr (%)	B	100 ± 8	0.308	−0.40	96 ± 8	0.631	−0.12	98 ± 6	0.029 *	0.66	0.606	0.01	
	A	100 ± 4			98 ± 4			94 ± 12			0.020 *	0.10	P-W *
HECOR RT (ms)	B	429 ± 46	0.182	0.40	393 ± 55	0.603	0.11	414 ± 40	0.002 *	0.74	0.198	0.04	
	A	409 ± 31			396 ± 39			383 ± 41			0.167	0.05	
HECOR MT (ms)	B	243 ± 41	0.820	−0.08	220 ± 39	0.021 *	−0.49	234 ± 71	0.241	−0.28	0.119	0.06	
	A	251 ± 53			242 ± 46			245 ± 54			0.795	0.01	
SPANT RT (ms)	B	641 ± 190	0.007 *	0.77	585 ± 106	0.068	0.39	584 ± 109	0.297	0.25	0.437	0.02	
	A	597 ± 128			544 ± 115			566 ± 192			0.348	0.03	
SPANT MT (ms)	B	238 ± 80	0.169	−0.42	228 ± 45	0.100	−0.35	236 ± 52	0.764	−0.07	0.558	0.02	
	A	239 ± 69			240 ± 55			236 ± 58			0.576	0.02	
SPANT cr (%)	B	95 ± 25	0.107	−0.67	95 ± 10	0.420	−0.21	95 ± 12	0.438	−0.22	0.442	0.02	
	A	95 ± 10			95 ± 5			95 ± 10			0.729	0.01	
PUT n (ms)	B	2192 ± 826	0.095	0.50	1858 ± 351	0.256	0.25	1963 ± 329	0.012 *	0.59	0.053	0.08	
	A	1870 ± 752			1698 ± 481			1733 ± 409			0.150	0.05	
PUT k (ms)	B	1283 ± 282	0.182	0.40	1245 ± 158	0.658	0.10	1241 ± 181	0.077	0.42	0.482	0.02	
	A	1239 ± 247			1150 ± 232			1207 ± 200			0.523	0.02	
PUT cr (%)	B	96 ± 2	0.406	−0.26	96 ± 4	0.925	0.03	97 ± 4	0.748	0.10	0.614	0.01	
	A	98 ± 8			96 ± 2			96 ± 4			0.468	0.02	
PAMT cr (%)	B	78 ± 11	0.176	−0.42	89 ± 11	0.646	−0.11	89 ± 8	0.168	0.36	0.024 *	0.10	P-W *
	A	83 ± 11			83 ± 14			83 ± 14			0.550	0.02	

SIRT—Simple Reaction Time; CHORT—Choice Reaction Time; HECOR—Hand–Eye Coordination Test; SPANT—Spatial Anticipation Test, PUT—Perception and Attention Test; PAMT—Anticipation Test; RT—Reaction Time; MT—Movement Time; cr—correct responses; n—neutral visual stimuli; k—critical visual stimuli; B—Before; A—After; Me ± IQR—Median ± Interquartile range; $p_{w}$—probability of testing (Wilcoxon test); $p_{kw}$—probability of testing (Kruskal–Wallis test); r—rank-sum correlation coefficient; $\eta^{2}$—effect size; p-h—post hoc; P-W—differences between pivot and wing player; *—statistical significance.

**Table 4:** Evaluation of the association between psychomotor abilities, training experience, and participation in the Super League.

B			A		
Variable	Training Experience	Super League Playing	Variable	Training Experience	Super League Playing
		Experience			Experience
SIRT RT (ms)	−0.07	0.11	SIRT RT (ms)	−0.10	−0.01
SIRT MT (ms)	0.16	0.28 *	SIRT MT (ms)	0.27 *	0.17
CHORT RT (ms)	0.01	−0.01	CHORT RT (ms)	−0.09	0.08
CHORT MT (ms)	0.17	0.23 *	CHORT MT (ms)	0.15	0.07
CHORT cr (%)	−0.05	−0.03	CHORT cr (%)	−0.03	0.06
HECOR RT (ms)	0.03	0.05	HECOR RT (ms)	−0.14	−0.03
HECOR MT (ms)	0.20	0.26 *	HECOR MT (ms)	0.26 *	0.10
SPANT RT (ms)	0.01	0.12	SPANT RT (ms)	−0.16	0.01
SPANT MT (ms)	0.06	0.01	SPANT MT (ms)	0.12	−0.02
SPANT cr (%)	0.14	−0.06	SPANT cr (%)	0.06	−0.12
PUT n (ms)	0.05	0.19	PUT n (ms)	−0.05	−0.03
PUT k (ms)	0.03	0.10	PUT k (ms)	−0.10	−0.17
PUT cr (%)	0.08	0.13	PUT cr (%)	0.14	0.15
PAMT cr (%)	−0.04	−0.17	PAMT cr (%)	0.16	0.19

B—Before; A—After; SIRT—Simple Reaction Time; CHORT—Choice Reaction Time; HECOR—Hand–Eye Coordination Test; SPANT—Spatial Anticipation Test, PUT—Perception and Attention Test; PAMT—Anticipation Test; RT—Reaction Time; MT—Movement Time; cr—correct responses; n—neutral visual stimuli; k—critical visual stimuli; *—statistical significance

## Data Availability

The original contributions presented in this study are included in the articlel. Further inquiries can be directed to the corresponding author.
